# T3SS effectors in Vibrios: Homology in sequence, diversity in biological functions?

**DOI:** 10.1080/21505594.2018.1435965

**Published:** 2018-04-18

**Authors:** Carlos R. Osorio

**Affiliations:** Departamento de Microbioloxía e Parasitoloxía, Instituto de Acuicultura, Universidade de Santiago de Compostela, Santiago de Compostela, Spain

**Keywords:** apoptosis, effector, T3SS, *V. alginolyticus*, virulence, VopS, VopQ

*Vibrio alginolyticus* is a Gram-negative bacterium that thrives in marine environments, and is also an important pathogen that causes vibriosis in a variety of marine animals. Infections in cultivated fish and invertebrates result in significant financial losses for aquaculture industries worldwide [1-3]. Furthermore, this bacterium can also be an opportunistic pathogen for humans, causing diarrhea as well as extraintestinal infections such as otitis and wound infections [4-6]. The mechanism of pathogenesis of this bacterium has not been completely elucidated but a number of virulence factors have been described, including an alkaline serine protease [7-8], hemolysins [9], siderophores used as host iron scavengers [10] and a type III secretion system [11].

The type III secretion system (T3SS) is a conserved apparatus among several Gram-negative bacteria that delivers effector proteins directly into host cells [12]. Effector proteins can behave as virulence factors that manipulate host cell physiology and cause a diversity of cellular responses and cell damage. T3SS have been characterized in a number of *Vibrionaceae* species. The first report of a T3SS in *Vibrio* was in *V. parahaemolyticus* where two sets of T3SS gene clusters, T3SS1 and T3SS2, were discovered [13]. T3SS1 is found in all *V. parahaemolyticus* strains, while T3SS2 is present only in KP-positive strains. Although initially thought to be absent from *V. cholerae* T3SS gene clusters were subsequently reported in non-O1, non-O139 strains [14,15] and in non-toxigenic O1 *V. cholerae* [16]. Similar clusters were identified in other *Vibrio* species, including *V. alginolyticus *[11,17], *V. mimicus *[18], *V. harveyi *[19] and *V. tubiashii *[17], among others. Recently, a T3SS was also reported in the type strain of another member of the *Vibrionaceae* family: the fish and human pathogen *Photobacterium damselae* subsp. *damselae* [20].

The best studied *Vibrio* T3SS so far is T3SS1 from *V. parahaemolyticus*, a pathogen for humans as well as for marine animals. This system induces death of mammalian cell lines in a multifaceted process that starts with autophagy, followed by cell rounding, and culminating with cell death in a caspase-independent process [21,22]. The effector protein VopQ is necessary and sufficient to induce autophagy in HeLa cells through its interaction with the V_o_ domain of the V-type H^+^-ATPase in lysosomal membranes [23,24]. Another effector, VopS, is involved in cell rounding and in the collapse of the actin cytoskeleton by inhibiting Rho GTPases [25].

The *V. alginolyticus* genome encodes a system homologous to the *V. parahaemolyticus* T3SS1, including homologues of VopQ and VopS. Interestingly, it was shown that the *V. alginolyticus* T3SS was responsible for causing rapid apoptosis, cell rounding and osmotic lysis in the fish cell line EPC [11]. These findings were in clear contrast with the effect of *V. parahaemolyticus* on mammalian cells which is characterized by the activation of autophagy but not of apoptosis [21,22]. Interestingly, when the same *V. alginolyticus* strain was used to infect the human HeLa cell line apoptosis was not induced but activation of autophagy occurred instead [26].

In the current issue of Virulence, Zhe Zhao and colleagues [27] uncovered the role of two *V. alginolyticus* T3SS effector proteins, Val1686 and Val1680, in apoptosis, cell rounding and osmotic lysis in the fish cell line FHM. The authors report the singularities of these two *V. alginolyticus* proteins, which are homologues of the *V. parahaemolyticus* T3SS1 VopS (91% id.) and VopQ (88% id.) effectors, respectively. Zhao and colleagues show here that *V. alginolyticus* VopS not only contributes to cell rounding of fish cells by inhibiting Rho GTPases but that it also is essential for the induction of apoptosis. A deletion mutant unable to produce VopS is severely impaired in its ability to induce these two cell responses and transfection of VopS into the fish cell line FHM is sufficient to cause cell rounding and apoptosis. The authors also show that the *V. alginolyticus* VopQ contributes to fish cell lysis but does not induce autophagy, a characteristic that distinguishes it from its *V. parahaemolyticus* homologue VopQ.

The findings reported by Zhao and colleagues in the present issue of Virulence provide new insights into the mechanisms of *V. alginolyticus* pathogenesis for fish cells. These data unequivocally demonstrate that this marine pathogen is capable of inducing apoptosis in fish cells and this effect is dependent on the T3SS effector VopS. Importantly, these results suggest that the type of host cell line used in the experiments is a variable of the maximal importance when drawing conclusions about the biological effects caused by a T3SS effector protein. The inherent differences between fish and mammalian cells may constitute the main reason why the *V. alginolyticus* and *V. parahaemolyticus* VopS and VopQ proteins induce distinct cellular responses. Very elegant experiments have contributed to unveil the roles of *V. parahaemolyticus* VopS and VopQ using mammalian cell lines and yeasts as host cell models [24,25,28,29], but information on the effects of these effectors in fish cells is scarce. In the light of the novel findings reported by Zhao and colleagues [27], it would be of high interest to compare the cellular effects caused by pairs of homologous T3SS effector proteins from different *Vibrio* species using the same host cell line. Future studies are needed in order to determine whether the different cellular responses elicited by *V. alginolyticus* and *V. parahaemolyticus* T3SS effectors are due to amino acid substitutions in homologous proteins or to inherent differences between fish and mammalian cells.
